# DeepMito: accurate prediction of protein sub-mitochondrial localization using convolutional neural networks

**DOI:** 10.1093/bioinformatics/btz512

**Published:** 2019-06-20

**Authors:** Castrense Savojardo, Niccolò Bruciaferri, Giacomo Tartari, Pier Luigi Martelli, Rita Casadio

**Affiliations:** 1 Biocomputing Group, Department of Pharmacy and Biotechnology (FaBiT), University of Bologna, Bologna, Italy; 2 Institute of Biomembranes, Bioenergetics and Molecular Biotechnologies (IBIOM), Italian National Research Council (CNR), Bari, Italy

## Abstract

**Motivation:**

The correct localization of proteins in cell compartments is a key issue for their function. Particularly, mitochondrial proteins are physiologically active in different compartments and their aberrant localization contributes to the pathogenesis of human mitochondrial pathologies. Many computational methods exist to assign protein sequences to subcellular compartments such as nucleus, cytoplasm and organelles. However, a substantial lack of experimental evidence in public sequence databases hampered so far a finer grain discrimination, including also intra-organelle compartments.

**Results:**

We describe DeepMito, a novel method for predicting protein sub-mitochondrial cellular localization. Taking advantage of powerful deep-learning approaches, such as convolutional neural networks, our method is able to achieve very high prediction performances when discriminating among four different mitochondrial compartments (matrix, outer, inner and intermembrane regions). The method is trained and tested in cross-validation on a newly generated, high-quality dataset comprising 424 mitochondrial proteins with experimental evidence for sub-organelle localizations. We benchmark DeepMito towards the only one recent approach developed for the same task. Results indicate that DeepMito performances are superior. Finally, genomic-scale prediction on a highly-curated dataset of human mitochondrial proteins further confirms the effectiveness of our approach and suggests that DeepMito is a good candidate for genome-scale annotation of mitochondrial protein subcellular localization.

**Availability and implementation:**

The DeepMito web server as well as all datasets used in this study are available at http://busca.biocomp.unibo.it/deepmito. A standalone version of DeepMito is available on DockerHub at https://hub.docker.com/r/bolognabiocomp/deepmito. DeepMito source code is available on GitHub at https://github.com/BolognaBiocomp/deepmito

**Supplementary information:**

Supplementary data are available at *Bioinformatics* online.

## 1 Introduction

Mitochondria are double-membrane bound organelles present in all Eukaryotic cells and performing very important biological functions, which include energy production, calcium signaling, regulation of cell metabolism and apoptosis (Poveda-Huertes *et al.*, 2017).

Mitochondria are endowed with their own genome, coding for only few proteins. The vast majority of proteins that are localized into mitochondria are instead encoded by the nuclear genome, synthesized in cytoplasmic ribosomes and subsequently translocated into the organelle by means of different mechanisms, the most well-characterized of which is based on the molecular recognition of specific targeting signals at the N-terminus of the nascent protein (Dudek *et al.*, 2013).

The mitochondrial outer membrane separates the interior of the organelle from the rest of the cell, while the inner membrane encloses the mitochondrial matrix. In turn, the two membranes are separated by the intermembrane space. The existence of such internal compartmentalization suggests that proteins localized in the different mitochondrial compartments are specialized to fulfill different tasks or functions: hence, knowing the precise location of a protein inside mitochondria is crucial for its accurate functional characterization (Martelli *et al.*, 2015).

In the past years, many computational methods could discriminate mitochondrial from non-mitochondrial proteins, taking advantage of machine-learning algorithms to detect highly specific targeting signals localized at the N-terminal region of the protein sequence (Bannai *et al.*, 2002; Emanuelsson *et al.*, 2007; Savojardo *et al.*, 2014; Fukasawa *et al.*, 2015; Savojardo *et al.*, 2015).

A substantial lack of experimental information constrained the discriminative capability of tools to a small number of compartments. Recently, the increasing amount of sequence data and the availability of richer experimental evidence, allowed the development of computational methods suited to predict protein subcellular localization at a finer grain. Currently, tools make it possible to discriminate sub-nuclear (Kumar *et al.*, 2014), sub-chloroplastic (Savojardo *et al.*, 2017; Wang *et al.*, 2015; Shi *et al.*, 2011) and sub-mitochondrial (Du and Li, 2006; Shi *et al.*, 2011; Du and Yu, 2013; Fan and Li, 2012; Kumar *et al.*, 2018; Lin *et al.*, 2013; Mei, 2012; Nanni and Lumini, 2008; Zeng *et al.*, 2009) localizations. When considering sub-mitochondrial compartments, only the method of Kumar *et al.* (2018) allows discriminating up to four different possible localizations (matrix, outer, inner and intermembrane regions). All the approaches rely on different types of global protein features extracted from sequence, including sequence composition, pseudo-amino acid composition, residue physicochemical attributes and/or evolutionary information extracted from multiple sequence alignments (MSAs).

Here, we describe DeepMito, a novel method for predicting sub-mitochondrial localization. DeepMito is based on artificial neural networks and it adopts the convolutional neural network (CNN) architecture to extract relevant patterns from primary features. DeepMito discriminates four different sub-mitochondrial compartments and our implementation outperforms the only method previously described (Kumar *et al.*, 2018), addressing the same task.

We optimized the CNN architecture of DeepMito adopting a non-redundant, rigorous cross-validation procedure performed on a new dataset comprising 424 highly curated protein sequences extracted from UniprotKB/SwissProt and endowed with experimental evidence for sub-mitochondrial localization. Cross-validation results on this dataset highlighted good performances with Matthews Correlation values ranging from 0.46 to 0.65, depending on the compartment. These values well compare with the results of Kumar *et al.* (2018), ranging from 0.42 to 0.51, when discriminating the same compartments. In addition, we retrained our CNN architecture on the same dataset previously adopted (Kumar *et al.*, 2018), and further confirmed the effectiveness of DeepMito, with performances overpassing the previously reported ones.

Finally, we analyzed the ability of DeepMito in performing genome-scale analysis. To this aim, we extracted a dataset of 1050 mitochondrial human proteins from the Cell Atlas section of the Human Protein Atlas resource (Thul *et al.*, 2017). Computed localizations were assessed towards the fraction of human mitochondrial proteins endowed with experimentally annotated GO terms for one of the sub-mitochondrial compartment. In this test, DeepMito shows a very high level of agreement with available experimental annotations (ranging from 93% to 100%, depending on the discriminated compartment).

## 2 Materials and methods

### 2.1 Datasets

#### 2.1.1 The SM424-18 dataset

The main dataset used in this study was derived from UniprotKB/SwissProt (release 2018_02). We first selected all non-fragment protein sequences with evidence at protein level and endowed with experimentally determined subcellular localization (evidence code ECO: 0000269) in one of the four sub-mitochondrial compartments: outer membrane (SL-0172), intermembrane space (SL-0169), inner membrane (SL-0168) and matrix (SL-0170). For sake of selecting the best possible set of annotations, proteins that are also localized in compartments other than mitochondria were excluded.

In order to obtain a non-redundant set of protein sequences, we performed clustering using the CD-HIT program (Li and Godzik, 2006) with global alignment and sequence identity threshold set to 40%. For each cluster generated by CD-HIT, we retained only the longest sequence.

After this filtering procedure, we ended-up with 424 mitochondrial proteins sharing at most 40% sequence identity computed at a global level. The dataset comprises 193 proteins from Metazoa, 166 from fungi, 60 from plants, 4 from Euglenozoa and 1 from Amoebozoa. Overall, the dataset comprises 74 outer membrane, 190 inner membrane, 25 intermembrane and 135 matrix proteins (Table 1).

**Table 1.** Summary statistics of the SM424-18 and the SubMitoPred datasets

Compartment	SM424-18^a^^,b^	SubMitoPred^b^^,c^
Outer membrane	74	82
Inner membrane	190	282
Intermembrane space	25	32
Matrix	135	174
Total	424	570

a This paper.

b Number of sequences.

c From Kumar *et al.* (2018).

On our dataset (SM424-18) we adopted a 10-fold cross-validation. In order to avoid any possible bias between training and testing, we applied the following clustering procedure to generate cross-validation sets. First, the 424 protein sequences were cross-compared running all-against-all pairwise blastp with e-value threshold set to 0.001. From blast output, we built a similarity graph where nodes are protein sequences and edges among pairs of nodes were added if at least one blast hit with more than 30% sequence identity was found (no coverage threshold was set). On this graph, single-linkage clustering was performed computing connected components. Finally, all proteins falling in the same cluster were assigned to the same cross-validation set. In this way, we eliminated any possible sequence identity bias among training and testing, confining any residual sequence redundancy (even occurring locally) in the same cross-validation set. SM424-18 is available for download at http://busca.biocomp.unibo.it/deepmito/datasets.

#### 2.1.2 The SubMitoPred dataset

SubMitoPred is a dataset previously introduced to train and test the most recent approach for sub-mitochondrial localization prediction, addressing a four-compartment discrimination (Table 1, SubMitoPred, Kumar *et al.*, 2018). According to the authors, SubMitoPred (available at http://proteininformatics.org/mkumar/submitopred/download.html) was derived from UniprotKB/SwissProt release 2014_10, selecting protein sequences with the following criteria:

- Full-length proteins (no fragments) with experimental existence evidence.
- Protein length > 50 residues.
- Experimental sub-mitochondrial subcellular localization, retaining only proteins localized into a single compartment.
- Dataset internal redundancy reduced at 40% sequence identity using CD-HIT.

Overall, the dataset comprises 570 mitochondrial proteins distributed in the four different sub-compartments (Table 1).

SubMitoPred contains more proteins than our dataset. Our dataset SM424-18 and the one generated by Kumar *et al*. (2018) share 238 common proteins. Of the remaining 332 included in the SubMitoPred dataset but not in SM424-18, 326 are not present in our dataset because they are not annotated with the experimental evidence code ECO: 0000269; six proteins were excluded because they are annotated as localized in multiple compartments.

For sake of comparison, when necessary and as previously described (Kumar *et al.*, 2018), we split the SubMitoPred set into five cross-validation subsets. According to Kumar *et al.* (2018), they performed cross-validation by randomly splitting the set of 570 proteins into five subsets. In this study, we performed two different cross-validation splits: (i) random split as described in Kumar *et al.* (2018) and (ii) random split after sequence clustering using blastp (applying the same procedure described in the previous section for our SM424-18).

#### 2.1.3 The Human Cell Atlas dataset

To assess the capability of DeepMito in performing genome-scale analysis, we here adopted a dataset extracted from the Cell Atlas section of the Human Protein Atlas project (Thul *et al.*, 2017). This resource provides a comprehensive catalog of subcellular localization of proteins in human cells derived from transcriptomics experiments or antibody-based image profiling techniques. By this, it provides experimental evidence for subcellular localization of 12 073 human proteins.

Here, we focused on the subset of 1074 proteins that are found to be localized into mitochondria (Cell Atlas does not provide for these proteins sub-mitochondrial localization). The Cell Atlas database assigns a label to each annotation, representing its quality. Four different labels are defined (in decreasing order of quality): Enhanced, Supported, Approved and Uncertain. For the 1074 mitochondrial proteins considered in this study, Enhanced and Supported annotations cover about 50% of the dataset (165 + 347 = 512), 506 annotations are Approved while only a small fraction are Uncertain (56 annotations). In order to obtain a sufficient number of sequences, here, we decided to retain all annotations available for mitochondrial proteins.

ENSEMBL gene identifiers for the 1074 mitochondrial proteins were mapped to UniprotKB entries: after this step we were able to map 1050 ENSEMBL identifiers to UniprotKB entries. Twenty-four ENSEMBL genes were excluded because of non-clear or multiple mapping to UniprotKB.

On this set, we extracted available Gene Ontology term information using the QuickGO website (https://www.ebi.ac.uk/QuickGO/). We focused only on annotations of the GO Cellular Component, endowed with experimental evidence (ECO: 0000269) and derived from any source databank. As a result, 179 out of 1050 gene products were annotated with GO terms that are equal to or descendants of any of the four mitochondrial compartments considered in this study: 19 proteins in mitochondrial outer membrane (GO: 0005741), 67 in mitochondrial inner membrane (GO: 0005743), 12 in mitochondrial intermembrane space (GO: 0005758) and 81 in mitochondrial matrix (GO: 0005759). Proteins that were annotated with GO terms related to multiple mitochondrial compartments were filtered-out form this set.

We refer to the full Cell Atlas dataset comprising 1050 protein sequences as Mito-CA-Full and to the subset of 179 annotated as Mito-CA-Annotated.

### 2.2 Feature descriptors

In this study, we considered three different feature types and evaluated their contribution (both individual and combined) to the prediction of protein sub-mitochondrial localization, when provided in input to the DeepMito convolutional network. In particular, the following features were considered:

- Residue one-hot encoding (SEQ), where each residue in a protein sequence is encoded using a 20-dimensional vector with all zero components except for the one representing the residue. Overall, each protein sequence is represented by a matrix with L rows and 20 columns, where *L* is the length of the sequence.
- Residue physical–chemical properties (PROP), where each residue in a protein sequence is encoded using the 10 different numerical values introduced by Kidera *et al.* (1985). These values derive from a multivariate statistical analysis of a set of 188 different properties of naturally occurring amino acids and can be used to compactly represent the physical–chemical nature of each residue. Overall, each protein is encoded with a matrix with L rows and 10 columns, where *L* is the length of the sequence.
- Evolutionary information, in the form of Position Specific Scoring Matrices (PSSM) as computed, for each sequence in the datasets, by running the PSI-BLAST (Altschul *et al.*, 1997) program against the Uniref90 dataset (release March 2018) for three iterations and e-value threshold set to 0.001. Overall, PSSM for a given protein is a matrix with L rows and 20 columns, where *L* is the length of the sequence. Internally, the program computes the matrix by considering the MSA obtained by (i) stacking all pairwise alignments between query and similar sequences found after each iteration and (ii) removing MSA columns corresponding to gaps in the query sequence. In this way, the PSSMs have always a number of rows that coincides with the length of the query sequence. Raw PSSM values extracted from the PSI-BLAST checkpoint file (generated by the program after each iteration) were mapped in the range [0-1] using a sigmoid function, defined as:
  (1) $$f\left(x\right)=\frac{1}{1+e^{-x}}$$

Feature descriptors are combined protein-wise with simple concatenation along the sequence axis. For instance, combining PSSM and PROP matrices of dimensions L×20 and L×10 leads to a L×30 matrix.

### 2.3 CNNs

CNNs have their main application domain in the Computer Vision area (LeCun *et al.*, 2015). Nevertheless, they have been proven to be very effective also for sequence analysis tasks in Genomics and Computational Biology, as highlighted by the increasing number of successful applications available in literature (Alipanahi *et al.*, 2015; Almagro Armenteros *et al.*, 2017; Angermueller *et al.*, 2016; Savojardo *et al.*, 2018).

In the context of bio-sequence analysis, inputs are routinely protein or a DNA sequences of variable length on which one wants to detect some feature or attribute, both at the level of individual residues (i.e. sequence labeling) or at a global level (i.e. sequence classification).

Each residue in a sequence is represented with low-level features, e.g. residue properties, one-hot encoding or sequence profiles. The number of features encoding for a given residues is referred to as channels in the CNN context.

A typical CNN is a feed-forward architecture comprising two different types of layers: convolutional and pooling layers. Formers are used to extract salient features from the input by means of filters or motif detectors, whose parameters are learnt during training and are used to scan the input. Pooling layers are instead parameter-free, and they are used for downsampling, namely to reduce the input dimensionality by selecting/aggregating the most relevant features extracted by convolutional layers according to some predefined function (e.g. average, max or min functions).

More formally, let be $X=\left(X_{1},\ldots ,X_{L}\right)$ an input sequence of length L where each $X_{i}\in R^{d}$ is a d-dimensional vector (i.e. d is the number of input channels). We restrict our attention to CNN architectures suited to sequence classification, i.e. the task of classifying the input sequence X into K different classes.

A convolutional layer is a collection $M=\left(M^{1},\ldots ,M^{F}\right)$ of F different motif detectors, each of which can be seen as a weight matrix of dimension w×d:

(2) $$M^{i}={\left[\begin{array}{ccc} M_{1,1}^{i} & \cdots & M_{1,d}^{i}\\ \vdots & \ddots & \vdots \\ M_{w,1}^{i} & \cdots & M_{w,d}^{i} \end{array}\right]}_{w\times d}$$

where w is the width of the motif and d is the number of input channels.

Using a sliding-window approach, the i-th motif detector produces an output sequence $\left(C_{i,\;1},\ldots ,C_{i,L}\right)$ having the same length L of the input and where each $C_{i,j}$ is computed as:

(3) $$C_{i,j}=g\left(\sum_{k=1}^{w}\sum_{r=1}^{d}M_{k,r}^{i}X_{j+k-\left\lfloor w/2\right\rfloor ,r}\right)$$

Sequence termini are handled by adding explicit zero-padding at the beginning and end of the input sequence X. In Equation (3), g is an activation function used to transform the raw motif score. Many different activation functions exist; however, routinely Rectified Linear Units (ReLUs) are adopted, defined as:

(4) $$g\left(x\right)=\max\left(0,x\right)$$

Overall, a convolutional layer endowed with F independent motif detectors compute an output matrix C with F rows and L columns:

(5) $$C={\left[\begin{array}{ccc} C_{1,1} & \cdots & C_{1,L}\\ \vdots & \ddots & \vdots \\ C_{F,1} & \cdots & C_{F,L} \end{array}\right]}_{F\times L}$$

Pooling layers following convolutions reduce the dimensionality of the matrix C along the second dimension (i.e. L). Different types of pooling are possible.

In *local pooling* with pool size p, an aggregating function t is computed row-wise over a set of p non-overlapping neighboring columns of C, transforming it into a new matrix $P^{\mathrm{local}}$ of dimension F×L/p:

(6) $$P^{\mathrm{local}}={\left[\begin{array}{ccc} t\left(C_{1,1},\ldots ,C_{1,p}\right) & \cdots & t\left(C_{1,L-p},\ldots ,C_{1,L}\right)\\ \vdots & \ddots & \vdots \\ t\left(C_{F,1},\ldots ,C_{F,p}\right) & \cdots & t\left(C_{F,L-p},\ldots ,C_{F,L}\right) \end{array}\right]}_{F\times L/p}$$

In *global pooling*, the pool size p is equal to L and the input matrix C is completely flattened into a column vector $P^{\mathrm{global}}$ of dimension F×1, computing the pooling function t row-wise over the entire set of L columns:

(7) $$P^{\mathrm{global}}={\left[\begin{array}{c} t\left(C_{1,\;1},\ldots ,C_{1,L}\right)\\ \vdots \\ t\left(C_{F,\;1},\ldots ,C_{F,L}\right) \end{array}\right]}_{F\times 1}$$

Common pooling functions are average, sum, maximum and minimum.

Overall, one or more successive applications of convolution-pooling layers transform the input X into a feature map P corresponding to the output of last pooling layer in the architecture. The feature map is then flattened into a single vector $v\;\in R^{m}$ (being m the total dimension of the feature map after the last pooling) and provided in input to a standard fully-connected network that first maps v into a hidden layer with H units $h_{1},\ldots ,h_{H}$ such that:

(8) $$h_{i}=a\left(\sum_{j=1}^{m}\omega_{j,i}^{h}v_{j}+b_{i}^{h}\right)$$

where $\omega_{j,i}^{h}$ and $b_{i}^{h}$ are the connection weights and bias of the i-th hidden unit, respectively, while the function a is an activation function (e.g. ReLU).

Finally, the hidden layer is mapped to the output layer comprising K units $o_{1},\ldots ,o_{K}$, one for each class, as follows:

(9) $$o_{i}=u\left(\sum_{j=1}^{K}\omega_{j,i}^{o}h_{j}+b_{i}^{o}\right)$$

where $\omega_{j,i}^{o}$ and $b_{i}^{o}$ are the connection weights and bias of the i-th output unit, respectively, and u is the output activation function, typically softmax or sigmoid, depending on the type of output desired.

In summary, a CNN architecture for sequence classification can be divided into two parts: the first part, where convolution-pooling layers are applied, is devised to feature extraction and selection; the second part, consisting in the final fully connected network, performs the actual classification of the sequence into K different classes.

### 2.4 The DeepMito CNN architecture

**Fig. 1.**
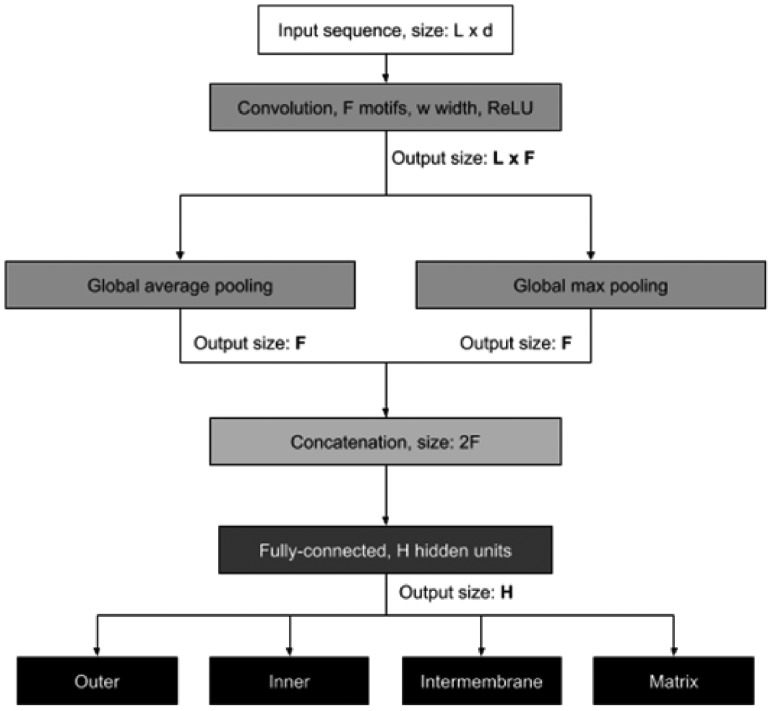
Schematic view of the DeepMito CNN architecture.

Prediction of protein sub-mitochondrial localization can be naturally defined as a multi-class sequence classification problem where each input protein sequence is classified as belonging to one of K=4 different mitochondrial sub-compartments. In this respect, DeepMito is based on the CNN architecture depicted in Fig. 1.

The input protein sequence where each residue is encoded as a d-dimensional vector (d varies according to the input feature considered) is scanned using a single convolutional layer comprising F detectors of width w (we tested several different values for these two variables, see next section for details). The convolutional layer output is then processed by two parallel pooling layers computing global average and maximum functions. The rationale behind this choice is to compute both the average motif signal and its peak along the input sequence, trying to capture different types of patterns.

The two pooling layers are then concatenated into a single vector and provided to the final classification network with H hidden units (also H has been optimized as detailed in the next section). Overall, the network has four independent output units with sigmoid activation function representing scores that quantify the membership of the input protein to each considered compartment: outer membrane, inner membrane, intermembrane space and matrix. The protein is predicted as localized into the highest-scoring compartment.

The DeepMito CNN was trained using a Stochastic Gradient Descent optimizer by minimizing the cumulative binary cross-entropy loss function. More formally, consider a training set $D={\left\{\left(X^{\left(i\right)},Y^{\left(i\right)}\right)\right\}}_{i=1,\ldots ,N}$, where $X^{\left(i\right)}$ is an input protein sequence while $Y^{\left(i\right)}$ is a 4-class target vector storing the membership of each protein to one of the compartments.

The cumulative binary cross-entropy loss function is defined as:

(10) $$E\left(D\right)=\sum_{j=1}^{K}E_{j}(D)$$

where each $E_{j}(D)$ is the binary cross-entropy loss function for class j and defined as:

(11) $$E_{j}\left(D\right)=-\frac{1}{N}\sum_{i=1}^{N}Y_{j}^{\left(i\right)}\log\left(o_{j}^{\left(i\right)}\right)+(1-Y_{j}^{\left(i\right)})\log\left(1-o_{j}^{\left(i\right)}\right)\;$$

where $o_{j}^{\left(i\right)}$ is the output of the CNN for the j-th class when the sequence $X^{\left(i\right)}$ is provided in input.

### 2.5 Model selection and implementation

A CNN architecture like the one depicted in Fig. 1 has several hyperparameters that need to be optimized. In particular, to define a convolutional layer we need to specify the number F of motif detectors as well as their width w. Analogously, for fully-connected layers, we need to optimize the number H of units in the hidden layers.

In order to find optimal hyperparameters, we defined a set of possible values for each hyperparameter (see Supplementary Table S1 for the complete list of tested values) and adopted the following grid-search procedure to search for their optimal combination. In particular, using the SM424-18 dataset, a complete 10-fold cross-validation was running for all possible combination of hyperparameters, using one of the subsets as testing set, eight subsets as training set and one as validation set (different from testing). Each individual training was running for 100 epochs starting with random initialization for all adjustable network weights. Early stopping on validation loss was used to prevent overfitting. We then selected, among all possible combinations of parameters, the one achieving the highest performance on validation data. We used the Generalized Correlation Coefficient (GCC) (Baldi *et al.*, 2000) index to compare different architectures (see next section for the formal definition of the GCC). The optimal set of hyperparameters was then frozen and used to score the CNN on testing data.

DeepMito was implemented in Python 2.7 and using the Keras v. 2.2.4 (https://keras.io) deep-learning library with Tensorflow v. 1.11 (https://www.tensorflow.org) as backend.

### 2.6 Scoring performance

Performances of our method were scored by computing the multi-class confusion matrix M where $M_{i,j}$ is the number of proteins belonging to class i and predicted in class j.

Single-class predictions were scored using the Matthews’ Correlation Coefficient (MCC) for each class k, defined as:

(12) $$\mathrm{MCC}\left(k\right)=\frac{M_{k,k}n_{k}-o_{k}u_{k}}{\sqrt{\left(M_{k,k}+o_{k}\right)(M_{k,k}+u_{k})(n_{k}+o_{k})(n_{k}+u_{k})}}$$

where $o_{k}=\sum_{i\neq k}M_{i,k}$ is the number of over-predictions for the class k, $u_{k}=\sum_{i\neq k}M_{k,i}$ is the number of under-predictions for the class k and $n_{k}=\sum_{i\neq k}\sum_{j\neq k}M_{i,j}$ is the number of proteins correctly predicted as not being in class k (i.e. correct negative predictions with respect to class k).

Moreover, we adopted the GCC, described in Baldi *et al.* (2000), and providing a single measure to score classifications involving more than two classes. In particular, for each class k, we can compute the number $a_{k}\;$of proteins in class k as:

(13) $$a_{k}=\sum_{i=1}^{K}M_{k,i}$$

and the number $b_{k}$ of proteins predicted in class k as:

(14) $$b_{k}=\sum_{i=1}^{K}M_{i,k}$$

Then we define the following matrix e as:

(15) $$e_{i,j}=\frac{a_{i}b_{j}}{N}$$

where $N=\sum_{i=1}^{K}\sum_{j=1}^{K}M_{i,j}$ is the total number of proteins in a dataset.

The GCC is then defined as:

(16) $$\mathrm{GCC}=\sqrt{\frac{\sum_{i=1}^{K}\sum_{j=1}^{K}\frac{{\left(M_{i,j}-e_{i,j}\right)}^{2}}{e_{i,j}}}{N(K-1)}}$$

The GCC value ranges from −1 to 1 and a GCC equal to 0 corresponds to predictions no better than random.

## 3 Results

### 3.1 Assessing the contribution of the different features

In order to build an optimal predictor for protein sub-mitochondrial compartment prediction, we first assessed the predictive power of each type of feature. In this study, as already detailed in Section 2.2, three basic type of features were considered: protein primary sequence (encoded using the standard residue one-hot encoding), protein physical–chemical attributes and evolutionary information in the form of PSSMs.

These three basic feature types were then combined and provided in the input to the DeepMito CNN architecture. In doing this, we considered protein primary sequence and PSSMs as alternative choices, scoring them individually or combined with protein physical–chemical attributes. For each evaluated feature set, a different CNN has been trained in cross-validation on the SM424-18 dataset, optimizing network architecture as explained in Section 2.5. Performance scores are reported in Table 2.

**Table 2.** Cross-validation performance on the SM424-18 dataset using different feature sets

Feature set	MCC(O)^a^	MCC(I)^a^	MCC(T)^a^	MCC(M)^a^	GCC^b^
SEQ^c^	0.17	0.15	0.13	0.07	0.15
PROP^d^	0.17	0.07	0.22	0.13	0.19
PSSM^e^	0.51	0.47	0.42	0.57	0.50
SEQ+PROP	0.16	0.07	0.55	0.09	0.34
PSSM+PROP	0.46	0.47	0.53	0.65	0.54

a MCC (O, I, T, M): Matthews Correlation Coefficient of Outer, Inner, Intermembrane and Matrix localization, respectively.

b GCC: Generalized Correlation Coefficient (Equation (16)).

c Residue one-hot encoding.

d Residue physicochemical attributes.

e PSSM: Position Specific Scoring Matrix.

Our results indicate that both primary sequence and protein attributes, when taken alone, are not sufficiently informative and both lead to limited prediction performances, with protein attributes slightly outperforming primary sequence (GCCs are 0.19 and 0.15, respectively).

As expected, evolutionary information plays a major role in improving prediction performance. In fact, when considered alone, the PSSM input significantly improves prediction performance, leading to a generalized improvement observable in all scoring indices and, in particular in GCC, raising it up to 0.50. When PSSM is combined with protein attributes, performances further improve reaching 0.54 of GCC. We then adopted this feature set for DeepMito and for all subsequence analyses.

The optimal CNN architecture comprises 256 convolutional motif detectors of width 19 and 256 hidden units in the fully connected hidden layer (see Section 2.4 for details on the DeepMito CNN architecture).

### 3.2 Analyzing DeepMito predictions on the SM424-18 dataset

Having selected the best CNN architecture and features set, we analyzed in detail DeepMito predictions on the SM424-18 dataset. This allowed to highlight strengths and limitations of our method.

Two aspects were taken into consideration: (i) how prediction performance varies across different taxonomic kingdoms and (ii) how DeepMito performs on different types of membrane proteins, namely single-pass (SP), multi-pass (MP) and peripheral membrane (PM) proteins.

Concerning the first issue, performance scores obtained on the different subsets of animals, plants and fungi proteins are reported in Table 3. It is worth noting that these results were not obtained by retraining DeepMito on the individual subsets of proteins but simply by isolating cross-validation predictions corresponding to each taxonomic set.

**Table 3.** DeepMito prediction performance on proteins from different taxonomic kingdoms

Kingdom	MCC(O)^a^	MCC(I)^a^	MCC(T)^a^	MCC(M)^a^	GCC^b^
Metazoa (193^c^)	0.44	0.44	0.52	0.69	0.54
Viridiplantae (60^c^)	0.45	0.52	0.90	0.76	0.71
Fungi (166^c^)	0.49	0.52	0.37	0.59	0.50

a MCC (O, I, T, M): Matthews Correlation Coefficient of Outer, Inner, Intermembrane and Matrix localization, respectively.

b GCC: Generalized Correlation Coefficient (Equation (16)).

c Number of sequences.

Comparing results in Table 3 with overall performance scores (Table 2, last row), we can observe a substantial robustness of the method across the three different kingdoms. In particular, performances are stable on animals (GCC 0.54) and slightly lower on fungi (GCC 0.50). Interestingly, on plant proteins prediction performances are significantly higher, reaching a GCC of 0.71, 17 percentage points higher than the one obtained on the full dataset (0.54).

In Table 4, results focus on the prediction of the localization of mitochondrial membrane proteins (i.e. experimentally localized into inner and/or outer membranes). In particular, we analyzed prediction results with respect to available experimental information on membrane protein topology, more specifically separating SP proteins (spanning the membrane with a single transmembrane segment), MP proteins (endowed with multiple transmembrane segments) and PM proteins (physically associated to the membrane but not spanning it). Out of 264 membrane proteins included in SM424-18, we were able to retrieve from UniprotKB topological information for 227 proteins.

**Table 4.** DeepMito prediction performance on mitochondrial membrane proteins with respect to annotated membrane protein topology

Topology	*N*_P_ (*N*_O_+*N*_I_)^a^	$Q_{2}^{\mathrm{mem}}$ (%)^b^	MCC(O)	MCC(I)
SP	71 (31+40)	92	0.43	0.38
MP	94 (21+73)	98	0.47	0.49
PM	61 (6+55)	36	0.36	0.09

a *N*_P_ (*N*_O_+*N*_I_): number of membrane protein (outer and inner).

b $Q_{2}^{\mathrm{mem}}$: the fraction of proteins correctly predicted in either inner or outer membrane.

SP: single-pass membrane protein; MP: multiple-pass membrane protein; PM: peripheral membrane protein.

In Table 4, for each topology class, we report the total number of proteins (N_P_), the number of outer and inner membrane proteins (*N*_O_ and *N*_I_, respectively), the fraction of proteins correctly predicted in either inner or outer membrane ($Q_{2}^{\mathrm{mem}}$) and the MCCs for inner and outer membrane classes.

Results show that the stronger the transmembrane signal is along the sequence the higher is the ability of DeepMito to properly recognize these proteins and discriminate them from non-membrane ones: 98% and 92% of MP and SP proteins, respectively, are correctly predicted as belonging to membrane compartments (either inner or outer membrane). In contrast, only 22 out of 61 (36%), PM proteins are correctly localized into membranes. This suggests that PM proteins are endowed with features that are more similar to proteins of matrix and/or intermembrane space. Thirty-nine PM proteins are incorrectly classified into globular compartments: out of these, 32 are annotated in UniprotKB as residing in the matrix side of the membrane. Interestingly DeepMito correctly assigns 28 out of 32 PM proteins to the matrix compartment.

### 3.3 Comparing DeepMito with other approaches

Performing a comparison among different approaches for sub-mitochondrial localization prediction is a challenging task, mainly because different methods are trained/tested using different datasets and many of the methods presented so far are no more available via respective web servers. For these reasons, we decided to carry out a direct comparison between DeepMito and the most recent approach described for the same four-compartment discriminative task (SubMitoPred, Kumar *et al.*, 2018). Furthermore, it is the only method running (at the specified web URL reported in the reference paper) and providing training/testing dataset for downloading.

For sake of comparison, we trained and tested DeepMito using a 5-fold cross-validation on the SubMitoPred dataset, comprising 570 proteins localized into the four different compartments (Table 1). In particular, two different procedures were applied to perform cross-validation. In a first experiment, we applied exactly the same procedure as described by the SubMitoPred authors, namely, random splitting the set of 570 proteins into five subsets (results labeled as RS in Table 5). In this comparative benchmark, our method significantly outperforms its competitor in all MCC scores. Noticeably, DeepMito performances are more stable across the four different classes, suggesting that our method is able to better cope with class imbalance. Proteins localized in the intermembrane space, despite their low abundance (only 32 out of 570 proteins in the SubMitoPred dataset) are recognized very well by DeepMito, achieving an MCC score of 0.54 against the 0.19 reported by SubMitoPred.

**Table 5.** Performance comparison of different methods

Method	Cross-validation	MCC(O)	MCC(I)	MCC(T)	MCC(M)
SubMitoPred^a^	RS	0.42	0.34	0.19	0.51
DeepMito	RS	0.45	0.68	0.54	0.79
DeepMito	CL	0.42	0.60	0.46	0.76

a Results taken from Kumar *et al.* (2018).

RS=cross-validation performed by random splitting the dataset. CL=cross-validation performed confining any local similarity into the same cross-validation set (see Sections 2.2.1 and 2.2.2 for details).

To further confirm the ability of DeepMito to capture informative patterns from data, we carried out an additional experiment using a more stringent procedure to perform cross-validation (marked as CL in Table 5). In this experiment, cross-validation split was computed confining pairs of sequence sharing any residual local similarity in the same cross-validation set (as done for our SM424-18 dataset, see Sections 2.2.1 and 2.2.2 for details). As expected, results obtained by DeepMito are slightly worse than those achieved by random splitting (justifying the adoption of the more stringent similarity reduction procedure) but still significantly higher than those achieved by SubMitoPred in all MCC scores.

### 3.4 Scoring DeepMito on genomic-scale analysis

As a final test, we evaluated DeepMito on genomic-scale analysis using the human mitochondrial dataset extracted from the Cell Atlas database (see Section 2.1.3). In particular, DeepMito was trained using the entire SM424-18 dataset and predictions generated for all the 1050 proteins included in the Mito-CA-Full dataset.

**Fig. 2.**
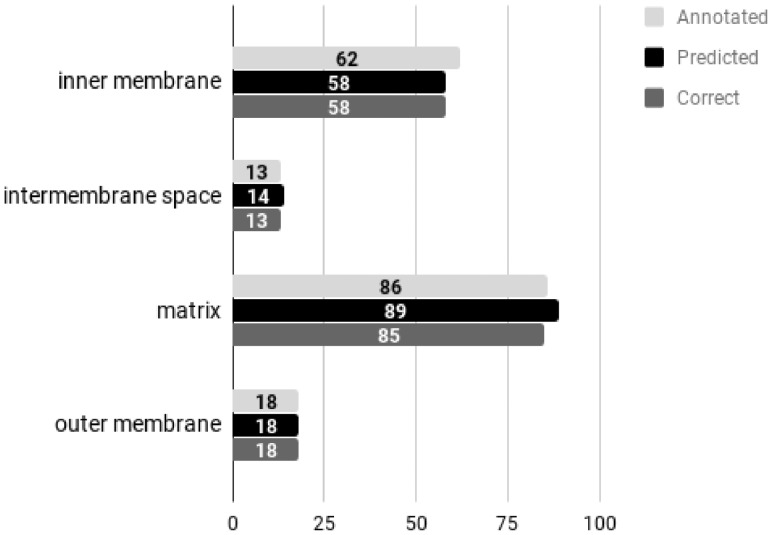
Distribution of annotations and DeepMito predictions on the Mito-CA-Annotated dataset.

First, we assessed DeepMito predictions with respect to available experimental annotations: for this, we extracted predictions on the Mito-CA-Annotated dataset comprising 179 sequences endowed with experimental GO terms relative to sub-mitochondrial compartments. Figure 2 summarizes distributions of annotations, DeepMito predictions as well as the number of correct predictions for each class. Evidently, DeepMito predictions correlate very well with available experimental evidence: overall, our method achieves a GCC of 0.97 on the Mito-CA-Annotated dataset.

**Fig. 3.**
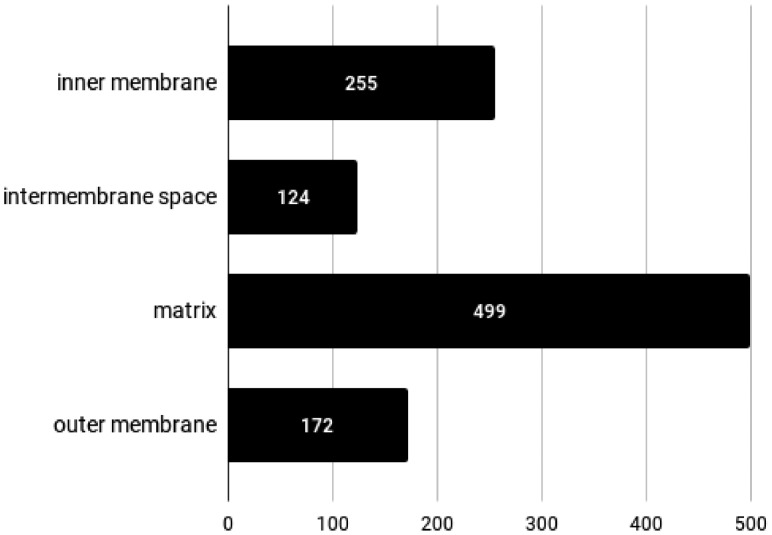
Distribution of DeepMito predictions on the Mito-CA-Full dataset.

In Fig. 3, we show the distribution of predicted classes for all the 1050 proteins in the Mito-CA-Full dataset. The relative abundances of predicted compartments are comparable to the ones observed in the Mito-CA-Annotated dataset. Complete results can be examined in detail at http://busca.biocomp.unibo.it/deepmito/hpa.

### 3.5 Software availability

We released DeepMito as web server at http://busca.biocomp.unibo.it/deepmito. The server supports the analysis of up to 200 sequences per submission: for each input protein, the server provides the predicted sub-mitochondrial compartment (as Gene Ontology Cellular Component term) along with a score associated with the prediction. Even though the server is intended to be used with proteins already known to be mitochondrial and for which the user is interested to know the precise localization inside the organelle, there is the possibility that users provide in input proteins that are not mitochondrial. In order to cope with this issue, the server performs a scanning of input proteins using two state-of-the-art predictors of mitochondrial localization: TPpred3 (Savojardo *et al.*, 2015), which predict mitochondrial localization by means of recognition of the targeting pre-sequence, and BaCelLo (Pierleoni *et al.*, 2006), which provide discrimination of mitochondrial proteins from proteins directed to other compartments. A protein is predicted as mitochondrial if at least one of the above methods classifies it as such. This piece of information is provided as additional output for the user.

We also provide a standalone version of the program implemented as a Docker container. The image is available on DockerHub at https://hub.docker.com/r/bolognabiocomp/deepmito. A tutorial on how to install and use the DeepMito docker container can be found at http://busca.biocomp.unibo.it/deepmito/software. DeepMito source code is also available on GitHub at https://github.com/BolognaBiocomp/deepmito.

## 4 Conclusions

DeepMito is a novel method for predicting protein sub-mitochondrial localization. Thanks to the power of a CNN architecture specifically designed to solve this task, DeepMito scores with good performances in different experiments aiming at testing its validity and applicability. In a four-compartment discrimination test, DeepMito scores higher than SubMitoPred, a recent method performing the same task. We tested DeepMito adopting a 10-fold cross-validation procedure on a newly developed training/testing set containing only mitochondrial proteins with location experimentally annotated. Then we also retrained and tested our predictor adopting the same set of proteins and the same cross-validation procedure of SubMitoPred, and again our method overpasses the state-of-the-art.

SubMitoPred is based on a combination of transfer-by-similarity and support vector machines. In contrast, DeepMito is based on artificial neural networks and it adopts the CNN architecture to extract relevant patterns from primary features. One immediate result is that our approach is robust with respect to class imbalance and provides very accurate predictions even for those compartments that are underrepresented in the training set (such as the intermembrane space, accounting for only few proteins).

The adoption of more complex architectures like recurrent layers may improve prediction performance in this task. However, in our experiments (data not shown), recurrent approaches lead to poor performance. This fact is maybe due to the scarcity of data which hampers proper training of complex architectures.

DeepMito well performs also on proteome-scale analysis, carried out on high-quality human proteins from the Cell Atlas database (Thul *et al.*, 2017). A present limit of DeepMito, due to the paucity of good quality available data is its present impossibility to predict multiple localization for a single protein sequence.

We propose DeepMito as a powerful and reliable tool for integration in functional annotation platforms.

## Funding

PRIN 2017 project 2017483NH8 (to C.S.) (Italian MIUR).

*Conflict of Interest*: none declared.

## Supplementary Material

btz512_Supplementary_Material
