# Supplementary material for: DeepMito: accurate prediction of protein sub-mitochondrial localization using convolutional neural networks
Source: Bioinformatics. 2019 Jun 20;36(1):56–64. doi: 10.1093/bioinformatics/btz512 (PMC6956790; doi:10.1093/bioinformatics/btz512)
Supplement: btz512_Supplementary_Material [file btz512_supplementary_material.docx]

**Supplementary Material**

DeepMito: accurate prediction of protein submitochondrial localization using convolutional neural networks

Castrense Savojardo^1^, Niccolò Bruciaferri^1^, Giacomo Tartari^1,2^, Pier Luigi Martelli^1,*^ and Rita Casadio^1,2^

^1^ Biocomputing Group, Department of Pharmacy and Biotechnology (FaBiT), University of Bologna, Italy. ^2^ Institute of Biomembranes, Bioenergetics and Molecular Biotechnologies (IBIOM), Italian National Research Council (CNR), Bari, Italy.

*To whom correspondence should be addressed.

**Table S1**. The complete list of parameters evaluated during training of the DeepMito CNN architecture.

| **Parameter** | **List of values evaluated** |
| --- | --- |
| Number of convolutional motifs (F) | 32, 64, 128, 256, 512 |
| Motif width (w) | 3, 5, 7, 9, 11, 13, 15, 17, 19, 21 |
| Number of hidden units in the fully-connected layer (H) | 64, 128, 256, 512, 1024 |
